# Notes and News

**Published:** 1889-05-25

**Authors:** 


### Notes and News.

Collected and Communicated.

Convalescent homes are throwing wide their doors, and patients are flocking to them, to enjoy this soft summer weather. There is a home at Heytesbury, Wilts, which receives ladies ; terms 25s. a week. There is a trained nurse attendance, and the home is one which our readers may be glad to remember, for it is suitable for cases for which few homes are provided.

The conversazione of the British Medical Society, held last week at the South Kensington Museum, was a brilliant affair. The guests, who were received by Mr. Brodie Sewell, president of the society, numbered nearly two thousand, and included most of the leaders in the medical world. There was some good music, both in the lecture-hall, where Mr. Ganz conducted a concert, and in the lower galleries, where the band of the Grenadier Guards performed.

Sir John Lubbock presided at the triennial festival of the Royal Maternity Charity, and in his speech said it stood the test of time as regarded its usefulness, and during its long existence had succoured more than half a million poor women in the hour of their greatest need. Last year it had assisted 3,600 mothers at a cost of £2,400, or less than 15s. a head, and he asked if it was possible to apply 15s. to a better purpose. But of this large number they had only eleven deaths, some of them due to other causes than child-birth, and this showed that the charity was useful, not only in preventing the injurious separation of mothers from their families, but in reducing the death-rate. In conclusion, he announced the receipt of a donation of 50 guineas from the Queen, and subscriptions to the total amount of about £500 were afterwards announced.

Sir R. N. Fowler, M.P., distributed the prizes in the faculty of medicine at University College last week. Among those present were Prof. John Erichsen (president), Sir George Young, Prof. Berkeley Hill, and Mr. J. T. Clance. The Dean (Prof. Marcus Beck) read the report, showing that during the year 340 students attended the classes for the medical faculty and the practice of the hospital, 99 being new students, an increase as compared with the previous year. Of a total of 207 who passed the various medical examinations of the University of London, 42, or 20 per cent., were from University College. Sir Robert Fowler then distributed the prizes. Gold medals were taken by Mr. T. L. Pennell, of London, in surgery, clinical medicine (Fellowes' medal), and clinical surgery (Liston gold medal); also by Mr. E. W. Selby, of Lewisham (physiology); Mr. H. A. Ballance, of London (anatomy); and Mr. L. E. Hill, of Reading (medicine).

In reply to a question in the House of Commons last week, put by Mr. Picton, Mr. Ritchie stated that he was not in a position to give the names of the Commissioners of the Vaccination Enquiry, but Lord Herschell had consented to be chairman. In accordance with the invariable practice of Royal Commissions, the proceedings will not be open to the public or to the Press.

The first annual report of the Victoria Infirmary of Glasgow, quite puzzled us at first; we looked in vain for the usual list of in and out-patients, result of cases, etc., and then we suddenly remembered that the infirmary is not yet nearly completed, and will not be opened till the autumn. The only fact we can give our readers, then, is that the income so far has exceeded £16,000, and it is a fact which is a credit to our Northern neighbours.

The *Electrical Review* has had the courage to expose some of the medical quacks of the day, who trade on the credulity of the public. But since the *Electrical Review* is not common in gullible circles, they have reprinted the letters and articles which appeared therein, and are circulating them as a pamphlet. The attack on C. B. Harness and his electropathic belts is well worth reading, and we wish more members of the medical profession would speak strongly on this subject. Anyone who is contemplating the purchase of a three-guinea belt which will cure all bodily ills under the sun, had better first procure and study this pamphlet.

The year's report on vivisection is published. It appears that the total number of experiments reported as having been performed by the fifty-five licensees was 1,069, being a decrease of 151 as compared with the total of 1887. Only eight experiments were reported as having been attended by pain ; the animals in these cases being rabbits, guinea-pigs, mice, and frogs. In the attainment of this gratifying result every assistance has been afforded by the licensees; who spare no trouble, by the use of anæsthetics, often applied locally, in addition to their internal administration and by the employment of strict antiseptic precautions, to secure freedom from suffering in the animals experimented upon.

There is great excitement at Bath, for Princess Louise, accompanied by the Marquis of Lorne, will visit that town on June 13 to open the new massage baths. The Princess is to leave Paddington at 10.30 a.m. by special train, which will stop at a temporary platform in the Sydney Gardens, instead of running on to the Great Western station. There is to be a civic luncheon in the Guildhall, after which the Princess will receive an address in the Pump Room, and she will then open the baths, and subsequently take a drive through the city, and leave by special train at 4.30 for Trowbridge, where she is to open a Jubilee Town Hall on the following day.

Many of our readers must have contributed to the sums of money which the Rev. Hugh B. Chapman, of St. Luke's, Camberwell, has been in the habit of sending out to Father Damien for the use of the lepers. Mr. Chapman now wishes all to remember that, though Father Damien is dead, the lepers are yet there, and he hopes the spirit of the martyr-priest yet lives in the hearts of those who watched with sympathy a noble life and death. He wishes that sums of £500 should be regularly forwarded to Father Damien's successor "for the benefit of the lepers under his charge and totally irrespective of creed.' In addition to this he desires to open a personal fund to the late Father Damien, to be disposed of by Cardinal Manning as his Eminence thinks fit. The money thus raised is to be spent in a memorial "to perpetuate the story of the heroism" of the devoted priest.

THE following vacancies are announced this week, the amount of the salary in each case being given after the name of the institution :

**Resident Medical Officers.**

Gloucester General Infirmary. House Surgeon.—£100 per annum, board, &c.
Adelaide Hospital, Australia. Medical Superintendent.—£500, board, &c.
Bolton Infirmary. Junior House Surgeon.—£100, board, &c.
Bristol Eye Hospital. Clinical Assistant.—£60.
Stafford Infirmary. Assistant to House Surgeon.—Board, &c.
Birmingham Children's Hospital. Assistant Medical Officer. £40, board, &c.
Dundee King's Cross Hospital. Medical Officer.—£300, board, &c.

**Non-Resident Medical Officers.**

Middlesex Hospital. Assistant Obstetric Physician.
Frome Union. Medical Officer.—£77.
Hampstead Consumption Hospital. Physician.
Sheffield Infirmary. Honorary Assistant Physician.

WHO can say that actors are not charitable? The Whittington Dramatic Society gave a performance of Mr. Sydney Grundy's "The Glass of Fashion" at St. George's Hall, on Tuesday evening, in aid of the Royal Hospital for Children and Women.—Miss Grace Hawthorne will play Mr. Richard Davey's "Paul and Virginia" at the Princess's on Thursday afternoon, June 27, in aid of the National Association for Employment of Reserve and Discharged Soldiers.—On Wednesday and Thursday evenings the Sick and Crippled Children's Home at Brondesbury was benefited by performances at the Kilburn Town Hall of a play called "For the Old Love's Sake," under the direction of Mr. and Mrs. Cecil Lamb.

---

ON May 11 the Marquis of Hertford opened the new wards of the Birmingham Orthopædic and Spinal Hospital. The extension, which has cost about £1,500, was carried out on the recommendation of the medical committee of the hospital, who reported in July, 1887, that the old premises were unsuitable for the purposes for which they were used. The additions and alterations have been carried out by Messrs. Sapcote and Sons, from plans prepared by the architects, Messrs. Osborne and Reading. In formally declaring the wing open, the Marquis of Hertford congratulated the friends of the hospital on the great improvement which had been effected, and hoped in time there would be money enough to fill the new wards, which were well designed.

---

PROF. HUXLEY has written a short autobiography for the May number of *Our Celebrities*, in which he describes his training and early work as a surgeon. His first visit to the post-mortem room nearly proved fatal to him, and is a warning to medical students generally. "I did not cut myself," he says, "and none of the ordinary symptoms of dissection poison supervened; but poisoned I was somehow, and I remember sinking into a strange state of apathy. By way of a last chance I was sent to the care of some good, kind people—friends of my father's—who lived in a farmhouse in the heart of Warwickshire. I remember staggering from my bed to the window on the bright spring morning after my arrival and throwing open the casement. Life seemed to come back on the wings of the breeze; and to this day the faint odour of wood-smoke, like that which floated across the farmyard in the early morning, is as good to me as the 'sweet south upon a bed of violets.' I soon recovered; but for years I suffered from occasional paroxysms of internal pain, and from that time my constant friend, hypochondriacal dyspepsia, commenced his half-century of co-tenancy of my fleshly tabernacle."

THE new children's ward to Worthing Infirmary has been opened, and all the necessary furniture has been given by friends. The ward contains eight cots, and is a useful and pretty addition to the building. The matron, Miss Jury, and the secretary, Mr. R. Grevett, are to be congratulated on the completion of a work for which they have worked indefatigably.

---

AN interesting meeting of the Street Ambulance Committee of the Hospitals Association took place at St. Bartholomew's last week. Twelve different litters were on view, and the committee had invited numerous medical and surgical celebrities to help them to decide on the comparative merits of the different ambulance carriages. After a careful examination, Sir Thomas Crawford and the other surgeons present expressed a strong opinion in favour of the Ashford litter. The committee now propose to proceed at once to order a sufficient number of litters to enable them to place one within an area of 500 yards of every part of London within the four miles district. Contributions in aid may be sent to the chairman of the committee, Sir Sydney H. Waterlow, St. Bartholomew's Hospital; or to the treasurer, Mr. H. L. Bischoffsheim, Throgmorton-street, E.C.

---

THE Church is often supposed to be too conservative in its tendencies, but this cannot be said of the Rev. E. S. Carpenter, of St. Chad's, Shrewsbury, who has just refused to sanction a very beautiful old ceremony. For over 140 years it has been the custom to hold the infirmary anniversary at St. Chad's. On these occasions the offertory has always been taken at the doors by a number of ladies. This characteristic feature of the service has now become the cause of a change, and the service is to be transferred from St. Chad's to a church where it can be perpetuated in the old form. Shrewsbury is an old town full of antiquarian interest, and we hope the infirmary will long benefit by the appreciation its inhabitants have for ancient customs. We congratulate Major-General Herbert on having done his best in trying circumstances to preserved the old ceremony.

---

THE Dean of Rochester, on Hospital Sunday, gave a word of praise to the medical profession which is a notable sign of the times. In the course of his remarks the Dean said that in all ages and countries, in all times and climes, the science and art of healing, the wisdom of the physician, the skill of the surgeon, have been admired and respected, not only from personal motives, but because they found so much to esteem in those who attended the sick; so much self-sacrificed labour, benevolence, and gentleness. In a most interesting pamphlet published by Surgeon Gordon on medicine in India, it was stated that the Arabs had, thousands of years ago, their officers of health in their villages; a place of reception for sick travellers, and even one for their sick animals. They also made great progress in medicine, and had specialists in those days performing successfully difficult surgical operations; the physician was the most honourable of all among them and required not only natural ability but moral character. They forbade intemperance, marriages between near relatives, and those with specified diseases, such as consumption, they would not allow to marry. It was the same in Greece and in Asia. So it was and had been among the Egyptians. The priests were doctors and were as a class connected with the art of healing and religion. It was most desirable it should be so now, and one was most thankful that so many missionaries were going out to India with a knowledge of medicine. He recommended the young people to attach themselves to the St. John's Ambulance Corps, so that they in cases of sudden disaster such as drowning or broken limbs, would be able to save their fellow creatures from intense pain and very often save life itself.

